# Preparation and application of an economical and environmentally friendly hydrate inhibitor in gas field development

**DOI:** 10.1371/journal.pone.0307109

**Published:** 2024-07-18

**Authors:** Dong Liao, Gui Su, Lei Liang, Jian He, Haifeng Ye, Qinghua Xiao, Yujia Xiong, Dong Wang, Lang Liu, Xingyu Luo

**Affiliations:** 1 CCDC Geological Exploration and Development Research Institute, Chengdu, China; 2 Sichuan Hengyi Petroleum Technology Service Co. Ltd., Chengdu, China; China University of Petroleum Beijing, CHINA

## Abstract

The prevention and control of natural gas hydrates is an important link in ensuring winter production. Traditional thermodynamic inhibitors, like methanol, are commonly utilized due to their low unit costs and pricing, but they come with considerable safety issues when used on-site due to their high toxicity, flammability, and explosive potential. A cost-effective and eco-friendly hydrate inhibitor was created by combining light polyol amine with other ingredients to solve this problem. At a concentration of 30%, the product has a flash point greater than 80°C and a solidification point of -45°C. With success rates of 99% and 100%, respectively, it was used for winter casing pre-injection anti-freezing operations and balancing tank defoamer anti-freezing operations. Experiments have demonstrated the effectiveness of this inhibitor in preventing the formation of natural gas hydrates. In wintertime on-site anti-freezing activities, the projected cost can be substituted for methanol, as it is essentially equivalent to methanol.

## 1. Introduction

**Fig 1:**
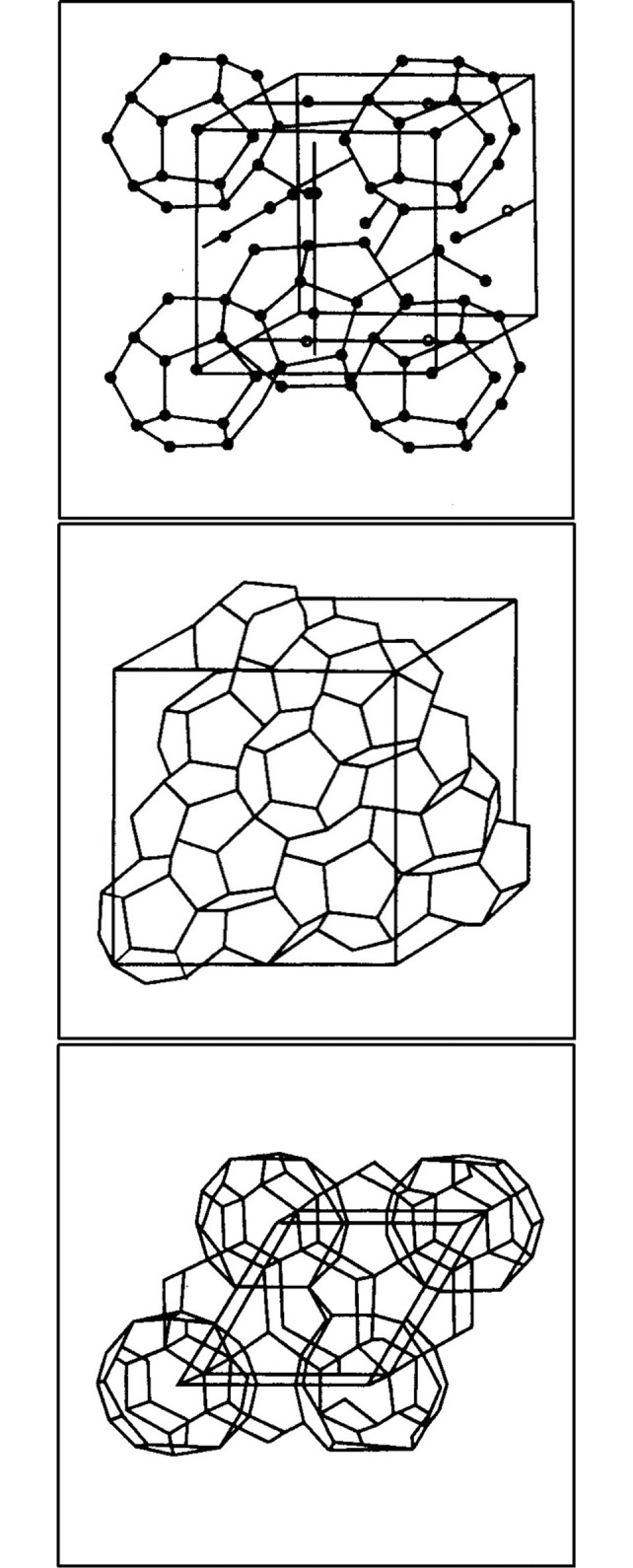
Three structures of natural gas hydrate [6]. (a: Type Ⅰ, b: Type Ⅱ, c: Type H.).

Natural gas hydrate is generated during natural gas extraction, transportation, and production [1, 2]. At a certain temperature and pressure, some small molecules in natural gas (N_2_, CO_2_, CH_4_, C_2_H_6_, C_3_H_8_, and etc.) can form crystal structures with water. Its structural characteristic is that gas molecules host in the lattice cage structure of crystalline water [3–5]. According to the different sizes and numbers of internal crystal pores, natural gas hydrate have three basic crystal structures: the cubic core structure (*Type I*), the rhombic cube structure (*Type II*), and the hexagonal structure (*Type H*). The structures were shown in Fig 1 [6].

*Type I* hydrate is mainly composed of methane hydrate and ethane hydrate, and with a cubic crystal structure [7]. It is the most widely distributed natural gas hydrate in nature. In this hydrate, gas molecules are fixed in a cage structure composed of water molecules, and their stability mainly depends on temperature and pressure conditions.

*Type II* hydrate is a rhombic crystal structure composed of alternating hexagonal and octagonal grids formed by water molecules [8–10]. There is a natural gas molecule in the center of the six membered ring, and two natural gas molecules around the eight membered body. This type of structure is suitable for natural gas hydrates in shallow marine sediments and terrestrial sediments.

*Type H* hydrate is a relatively new type of structure composed of a twelve membered ring grid, which is formed by water molecules and an eight membered grid arranged alternately [11, 12]. There is a natural gas molecule in the center of the twelve membered ring, and two natural gas molecules around the eight membered body. This type of structure is relatively stable under high pressure and low temperature conditions, and is suitable for natural gas hydrates in deep-sea sediments.

Caption credit: Sloan E D. Clathrate hydrates of natural gases (second ed.). Marcel Dekker, New York. 1990.

These structural types are not independent of each other, but can be transformed from one another. Under certain temperature and pressure conditions, one type of structure may transition to another [13–15]. However, without exception, these hydrates can clog the wellbore, pipelines, valves, and equipment, thereby affecting the normal operation of production [16–18]. It can be seen that the prevention and control of natural gas hydrates is of great significance for the normal production and transportation of natural gas.

At present, the prevention and control of natural gas hydrates are mainly divided into physical method and chemical method. The physical method mainly deprives the system of thermodynamic conditions for generating hydrates [19–21]. There are mainly heating method, pressure method, dehydration method, mechanical method, and etc. The most commonly used and effective method in the natural gas industry is chemical method [22–24]. Adding a certain dose of chemical inhibitors to the system can not only inhibit the formation of natural gas hydrates, but also dissolve the formed natural gas hydrates. According to the mechanism of action of inhibitors, they can be divided into kinetic inhibitors and thermodynamic inhibitors. Thermodynamic inhibitors change the thermodynamic conditions of the system (such as temperature, pressure, gas composition, and etc.), thereby altering the free energy of the hydrate phase, reducing the equilibrium constant of hydrate formation, and inhibiting hydrate formation [25–28]. Specifically, these inhibitors increase the free energy of the hydrate phase by reducing the gas partial pressure in the system, making it difficult to form hydrates. Common thermodynamic inhibitors include alcohols, salts, hydrocarbons, and etc.

Dynamic inhibitors mainly inhibit hydrate formation by slowing down the rate of hydrate formation [29–31]. These inhibitors slow down the formation and growth process of hydrate nuclei by increasing the resistance of gas molecule diffusion and changing the arrangement of water molecules, thus achieving the goal of inhibiting hydrate formation. Examples of several kinetic inhibitors: surfactants, polymers, nanomaterials, and etc.

In summary, the problems faced by kinetic inhibitors in their application are low inhibitory activity, poor universality, and significant influence from the external environment. Thermodynamic inhibitors mainly include alcohols and salts. The commonly used method for preventing and controlling hydrates on site is to inject excessive thermodynamic inhibitors to completely suppress the formation of hydrates in the borewell or pipeline. On site construction requires the consumption of a large amount of inhibitors, resulting in high costs and poor environmental protection.

In the winter extraction of natural gas, traditional thermodynamic inhibitor methanol is usually used for hydrate prevention and control operations. Methanol has the advantages of low unit cost and wide use, but it has the disadvantages of strong volatility, pollution of water and soil, flammability and explosiveness, and high transportation management costs. There are safety hazards in the field use. In order to solve the above problems, this paper develops a composite hydrate inhibitor with both economy and environmental protection, which replaces methanol in winter antifreeze operation to guarantee the normal exploitation of natural gas.

## 2. Experiments

### 2.1 Materials

Light polyol amine (LPA, ≥96%) and Methanol (MeOH, 99%) were purchased from Yasuda Chemical (Jiangsu) Co., Ltd., Jiangsu, China. Potassium acetate (KAc, 99%), Sodium chloride (NaCl), and Ethylenediaminetetraacetic acid disodium salt (EDTA, 99%) were purchased from Sinopharm Chemical Reagent Co., Ltd., Shanghai, China.

### 2.2 Preparation

Natural gas hydrate inhibitor (NGHI) was prepared by Response Surface Methodology (RSM). RSM is a visualization tool used to display the impact of two or more independent variables on the dependent variable [32]. It is commonly used to explore and optimize the optimal conditions in multi-variable systems. A four-factor, four-level response design was constructed to analyze the optimal mass ratios of the four inhibitor composites using the freezing point of the composite inhibitor at 50% concentration as the response indicator, and the factor levels are tabulated in Table 1.

**Table 1:** Design of factor levels.

No.	A(LPA)/g	B(KAc)/g	C(EDTA)/g	D(NaCl)/g
1	1	2	0.5	0.5
2	2	4	1.0	1.0
3	3	6	1.5	1.5
4	4	8	2.0	2.0

### 2.3 Measurements

The prepared inhibitor was added into water and stirred with a collector-type constant temperature heating magnetic stirrer at room temperature until completely dissolved, and a certain mass fraction of the solution was prepared. The flash point and freezing point were measured by Open Flash Point Tester and Freezing Point Tester (Shanghai Shenkai Petroleum & Chemical Equipment Co., Ltd., Shanghai, China).The interaction energy between molecules is calculated through GAMESS interface simulation.

## 3. Results

### 3.1 RSM

Composite inhibitors were prepared according to different ratios in Table 2, and the freezing points of inhibitors were measured at the concentration of 50%. The analytical response surface design was analyzed based on the data in Table 2.

**Table 2:** Freezing point of products under multi factor conditions.

No.	A(LPA)/g	B(KAc)/g	C(EDTA)/g	D(NaCl)/g	Freezing Point/°C
1	1	2	0.5	0.5	-34
2	1	4	1.0	1.0	-26
3	1	6	1.5	1.5	-30
4	1	8	2.0	2.0	-32
5	2	2	1.0	1.5	-27
6	2	4	0.5	2.0	-38
7	2	6	2.0	0.5	-46
8	2	8	1.5	1.0	-29
9	3	2	1.5	2.0	-31
10	3	4	2.0	1.5	-34
11	3	6	0.5	1.0	-41
12	3	8	1.0	0.5	-42
13	4	2	2.0	1.0	-35
14	4	4	1.5	0.5	-33
15	4	6	1.0	2.0	-39
16	4	8	0.5	1.5	-43

**Fig 2:**
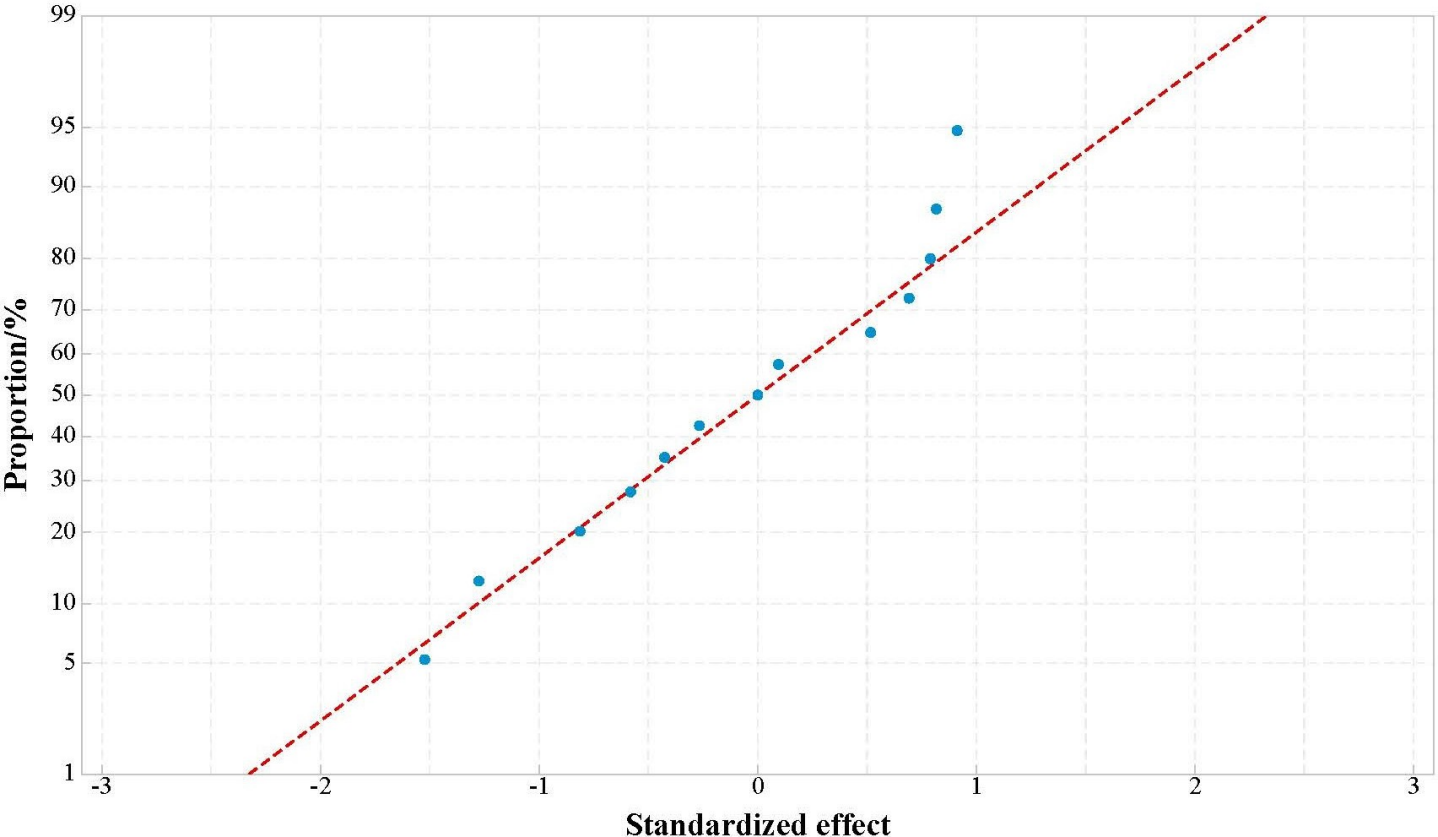
Proportion distribution of standardization effect.

Fig 2 shows the standardized probability distribution diagram of the response surface design. It can be analyzed that the standardized effect values of the response surface design are mainly distributed in the range of -1 to 1, and their distribution pattern is basically a straight line, satisfying the characteristics of normal distribution. This means that the distribution of data is consistent with the expected pattern of normal distribution [33]. The normal distribution is characterized by symmetry, i.e., the data points are uniformly distributed around the mean value, and most of the data points are concentrated near the mean value, while there are relatively few extreme values far from the mean value.

Further analysis was conducted on the response surface plots of response values at different levels of factors. Through the corresponding surface plots, the interaction between independent variables and how they collectively affect the dependent variable can be observed.

**Fig 3:**
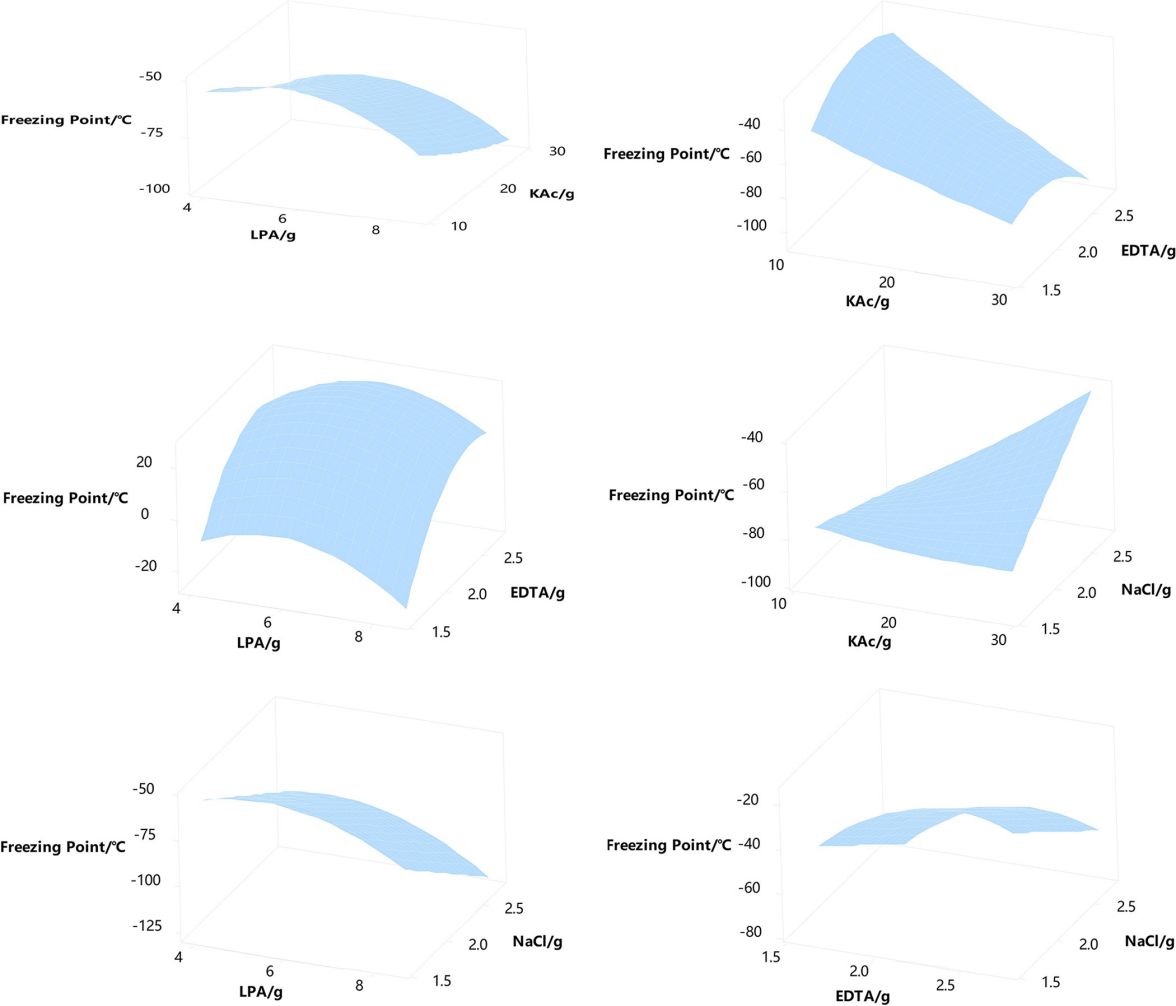
Response surface graph of freezing point under different factors.

Observe the shape of the surface graph, which is mainly a raised or flat surface. A raised surface graph usually indicates that the independent variable has a positive impact on the dependent variable, meaning that as the independent variable increases, the dependent variable also tends to increase, as shown in Fig 3A and 3B. A flat surface graph may indicate that the direct impact of the independent variable on the dependent variable is not significant, or may imply that there is no significant linear or nonlinear relationship between the independent and dependent variables within this specific data range (Fig 3E and 3F).

The optimal combination of independent variables was determined through response surface analysis: A: B: C: D = 2:4:1:1. And obtain the regression equation for freezing point (FP), and the variance between the values (R^2^) is 94.46%.


$$\begin{array}{c} FP=-242+25.6A-3.94B+211C-43.0D-2.44AA+0.024BB-43.6CC\\ +0.021AB+2.44AC-1.72AD-1.67BC+2.89BD \end{array}$$

Therefore, the optimal formulation of Natural gas hydrate inhibitor (NGHI) is as follows: 2g of LPA, 4g of KAc, 1g of EDTA, and 1g of NaCl. Substituting them into the regression equation, it is predicted that the freezing point of the inhibitor in this combination is about -85°C, which has good hydrate inhibition ability.

### 3.2 Performance

The freezing point and flash point of methanol and NGHI at different concentrations were comparatively analyzed in Table 3. The performance of inhibitors is usually judged by their freezing point, while the flash point reflects their safety in storage and usage. Under the same conditions, inhibitors have lower freezing points and higher flash points, with a freezing point of 30% NGHI close to that of 50% methanol. So it can reduce the cost of use and the safety of storage.

**Table 3:** Performance comparison analysis of methanol and inhibitor.

Concentration /wt.%	Freezing point/°C		Flash point/°C	
	MeOH	NGHI	MeOH	NGHI
10	10	-10	8	95
20	0	-22	5	91
30	-15	-45	3	86
40	-40	-71	1	81
50	-48	-82	-1	76

**Fig 4:**
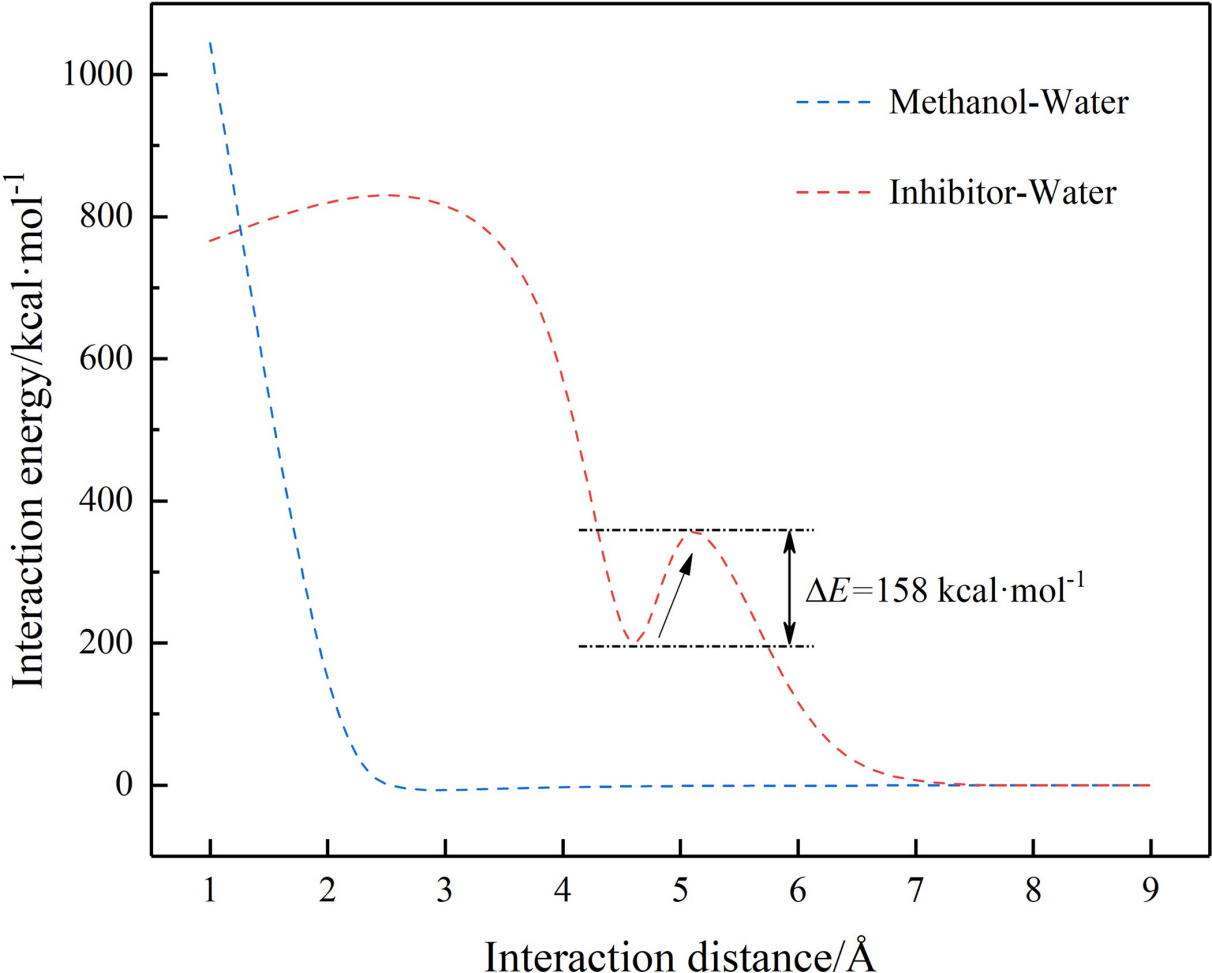
Interaction energy curves under different modes.

Through molecular simulation, the interaction energies between methanol and inhibitor and water molecules were calculated separately. The results are shown in Fig 4. The results show that methanol and water exhibit common mutual attraction, so the process of using methanol as an inhibitor mainly manifests as thermodynamic control. There is an oscillating peak between the inhibitor molecule and the water molecule in the range of 4–6 Å, with an interaction energy potential of 158 kcal·mol^-1^.

Among the inhibitors, LPA belongs to the category of kinetic inhibitors, while the rest belong to the category of thermodynamic inhibitors. Dynamic inhibitors mainly inhibit the formation of hydrates by slowing down the rate of hydrate formation. They are an effective hydrate inhibitor that slows down the formation and growth process of hydrate crystal nuclei by changing the arrangement of water molecules and increasing the resistance of gas molecule diffusion.

### 3.3 Application

#### 3.3.1. Casing pre-injection

Pre-injection anti-freezing operation of casing refers to the injection of foaming agent, including dilution water, at the pressure gauge of the Christmas tree casing, followed by the injection of NGHI. On the one hand, the inhibitor can replace the foaming agent and enter the annular space of the oil sleeve. On the other hand, the inhibitor can stay at the pressure gauge of the Christmas tree sleeve to the four-way of the casing. Ensure that this pipeline will not freeze due to the formation of hydrates, and reserve a channel for the next injection of foaming agent.

The test selected the measure wells in the same well area that carried out the soak and drain process, totaling 194 to carry out casing pre-injection anti-freezing operation. The implementation details are shown in Table 4.

**Table 4:** Anti-freezing situation of casing pre-injection.

Pressure/MPa			Temperature/°C		
High	Low	Average	High	Low	Average
12.98	1.15	4.96	-2.3	-27.3	-11.5
Injection /L·time^-1^	Injection times	Total volume/L	Blocking times	effective injection	Efficiency /%
5	2165	10825	9	2156	99.58

The effectiveness of this new environmentally friendly hydrate dissolver has been very reliable on typical single wells, both multiple operated wells and wells with high casing pressures. Plugging refers to plugging during the current injection of foaming agent after the last casing pre-injection, indicating that the last casing pre-injection failed to prevent the formation of hydrates. In the whole block, there were only 9 blockages in more than 2000 times of casing pre-injection anti-freezing operations. The success rate is more than 99%, reaching the same effect of methanol construction in previous years.

#### 3.3.2. Balance tank anti-freezing

Anti-freezing of defoamer in balance tank refers to adding a certain amount of anti-hydrate agent into the balance tank to ensure that the antifoam diluent is always not frozen in order to maintain the 24-hour drip state. Therefore, the whole process has to go through the coldest time of the day and night, and at the same time, the defoaming process is related to the thoroughness of the downstream water and gas separation, the importance of which is self-evident.

Due to the balance of the tank antifoam agent antifreeze single consumption of a larger amount of medicine (20L-50L, methanol is also so), and is a daily must add, taking into account the amount of medicine, from the whole region of the balance of the tank at various places randomly selected 4 places for the test. Part of the experimental data are shown in Table 5.

**Table 5:** Balance tank anti freezing medication dosage.

Day	Temperature/°C		Injection Volume/L		
	Low	High	Defoamer	Water	NGHI
1	2	-12	10	44	36
2	-4	-15	10	44	36
3	-1	-15	10	53	27
4	3	-12	10	53	27
5	0	-10	12	72	36
6	4	-11	12	72	36
7	0	-13	15	100	50
8	4	-10	15	100	50

The experiment showed that the defoamer diluent did not freeze in the equilibrium tank for 24 hours, and there was no dripping blockage phenomenon during 54 days of continuous operation. The defoaming of the four downstream pipelines involved was normal. For equivalent comparison, data statistics were conducted on balance tanks that still used methanol antifreeze at other locations during the same period. It was calculated that every 1.27L of hydrate solvent can effectively replace 1L of methanol for antifreeze operations in balance tanks.

According to the on-site test results, it can be seen that in the pre-injection anti-freezing operation of the casing and the anti freezing operation of the balance tank defoamer, the new environmentally friendly hydrate dissolution agent can replace methanol to inhibit the formation of hydrates.

### 3.4 Economics

Table 6 provides a cost comparison between a new environmentally friendly hydrate solvent and methanol. Prices are converted based on past year data using real-time exchange rates. Transportation of hazardous chemicals refers to the cost of renting a methanol transport vehicle. Storage of hazardous chemicals includes the cost of specialized warehouses for hazardous chemicals, equipped with fire extinguishers, alarms, sprinkler systems, detectors, and other safety facilities.

**Table 6:** Economic cost accounting.

Content	NGHI	MeOH
Materials/$·t^-1^	1,560	450
Transportation of hazardous chemicals/$·year^-1^	-	150,000
Storage of hazardous chemicals/$·year^-1^	-	56,000
Dosage/t·year^-1^	150	150
Total/$·year^-1^	234,000	273,500
Average/$·t^-1^	1,560	1,823

According to the cost comparison between NGHI and methanol, calculated based on one year’s usage, NGHI saves 263 $·t^-1^. NGHI can replace methanol to inhibit the formation of hydrates in winter antifreeze operations, solving the problems of flammable, explosive, and toxic methanol. It greatly improves the safety of transportation, storage, and use, reduces environmental pollution, and brings certain economic benefits.

## 4. Conclusions

1. Both indoor and on-site experiments have shown that NGHI has a lower freezing point, which can inhibit the formation of hydrates on site, ensure normal production on site in winter, and has good practicality.
2. Through equivalent comparative tests, the equivalent substitution ratio of NGHI and methanol in the pre injection anti freezing operation of the casing is 1:1, and the equivalent substitution ratio of hydrate dissolution agent and methanol in the anti freezing operation of the balance tank defoamer is 1.27:1. Through cost and usage estimation, the average cost and annual total cost of this new environmentally friendly hydrate dissolution agent and methanol are basically the same, indicating that it has good economic efficiency.
3. NGHI solves the problems of flammable, explosive, and toxic methanol, greatly improving the environmental and safety aspects of production, transportation, storage, and usage. It is expected to be widely promoted on site and has broad application prospects.
